# Fractal dimension to characterize interactions between blood and lymphatic endothelial cells

**DOI:** 10.1088/1478-3975/acd898

**Published:** 2023-06-12

**Authors:** Donghyun Paul Jeong, Daniel Montes, Hsueh-Chia Chang, Donny Hanjaya-Putra

**Affiliations:** 1 Bioengineering Graduate Program, University of Notre Dame, Notre Dame, IN 46556, United States of America; 2 Aerospace and Mechanical Engineering, University of Notre Dame, Notre Dame, IN 46556, United States of America; 3 Chemical and Biomolecular Engineering, University of Notre Dame, Notre Dame, IN 46556, United States of America; 4 Harper Cancer Research Institute, University of Notre Dame, Notre Dame, IN 46556, United States of America; 5 Center for Stem Cell and Regenerative Medicine, University of Notre Dame, Notre Dame, IN 46556, United States of America

**Keywords:** fractal dimension, morphogenesis, cell migration, blood and lymphatic endothelial cells

## Abstract

Spatial patterning of different cell types is crucial for tissue engineering and is characterized by the formation of sharp boundary between segregated groups of cells of different lineages. The cell−cell boundary layers, depending on the relative adhesion forces, can result in kinks in the border, similar to fingering patterns between two viscous partially miscible fluids which can be characterized by its fractal dimension. This suggests that mathematical models used to analyze the fingering patterns can be applied to cell migration data as a metric for intercellular adhesion forces. In this study, we develop a novel computational analysis method to characterize the interactions between blood endothelial cells (BECs) and lymphatic endothelial cells (LECs), which form segregated vasculature by recognizing each other through podoplanin. We observed indiscriminate mixing with LEC−LEC and BEC−BEC pairs and a sharp boundary between LEC−BEC pair, and fingering-like patterns with pseudo-LEC−BEC pairs. We found that the box counting method yields fractal dimension between 1 for sharp boundaries and 1.3 for indiscriminate mixing, and intermediate values for fingering-like boundaries. We further verify that these results are due to differential affinity by performing random walk simulations with differential attraction to nearby cells and generate similar migration pattern, confirming that higher differential attraction between different cell types result in lower fractal dimensions. We estimate the characteristic velocity and interfacial tension for our simulated and experimental data to show that the fractal dimension negatively correlates with capillary number (*Ca*), further indicating that the mathematical models used to study viscous fingering pattern can be used to characterize cell−cell mixing. Taken together, these results indicate that the fractal analysis of segregation boundaries can be used as a simple metric to estimate relative cell−cell adhesion forces between different cell types.

## 1. Introduction

During embryonic development, cells bound for different fates grow in close proximity to each other, and yet develop well-defined boundaries between separate tissues [1–3]. While undergoing rapid growth and differentiation, cells also retain their pattern forming abilities that drive morphogenesis and tissue formation [4–6]. Loss of cadherin function in embryogenesis, which is responsible for cell segregation, leads to a failure to form distinct compartments in the embryo and therefore causes embryonic lethality, highlighting the importance of cell−cell boundary formation in tissue morphogenesis [7, 8].

In order to achieve clear boundary formation, cells use a combination of differential adhesion, selective avoidance, and cortical tension to form boundaries [9, 10]. Differential adhesion is achieved through surface receptors which selectively recognize cells and form tighter bonds with them through the cadherin family of molecules [11–13]. Cells also develop selective repulsion through Eph-ephrin-guided mechanism, which has also been reported to play a role in cell segregation [14, 15]. Actomyosin also plays a role in cell segregation through mediation of cortical tension which affects cell cytosol to reshape boundaries [16, 17]. These factors contribute to the cells selectively forming tighter or looser bonds with surrounding cells, leading to a differential in affinity between cells of the same type and different type [9]. The degree of differential in affinity determines the crispiness of the borders between two cells types, with higher differential affinity leading to straighter borders [18]. Similar to two immiscible fluid−fluid surface, high degree of selective preference for cells of its own type leads to energetical unfavourability of rough, fuzzy surfaces and formation of straight borders to minimize the contact with cells of the same type [19].

This formation of straight, clear borders is important in stem cell-based tissue engineering, since stem cell-derived mature cells often suffer from immaturity and lower marker expression compared to their native mature counterparts [20–22]. Therefore, in tissue engineering applications requiring more than one type of cell, for example in vascularized tissue engineering involving the endothelial and target tissue-specific cells, the recognition ability of cells and the resulting differential affinity is crucial for free energy-driven self-organization of cells [23–26]. In order to predict the ability of two or more types of human induced pluripotent stem cell-derived cells to self-assemble into tissues, measuring the differential affinity between different cell types is crucial [2, 22]. Numerous studies have proposed mathematical models to explain the collective migration and organization behavior of cells [27]. A study by Mark *et al* proposed a dynamic instability model to predict cell−cell boundary formation which is solely affected by cell shape and motility without accounting for chemical gradients [28]. Another study by Kopf and Pismen proposed modeling of the cell−cell boundary as an elastic continuum that can respond to chemical and mechanical stimuli [29]. Here, we put forth our mathematical model to characterize cell−cell boundary inspired by the field of petroleum engineering.

**Figure 1.**
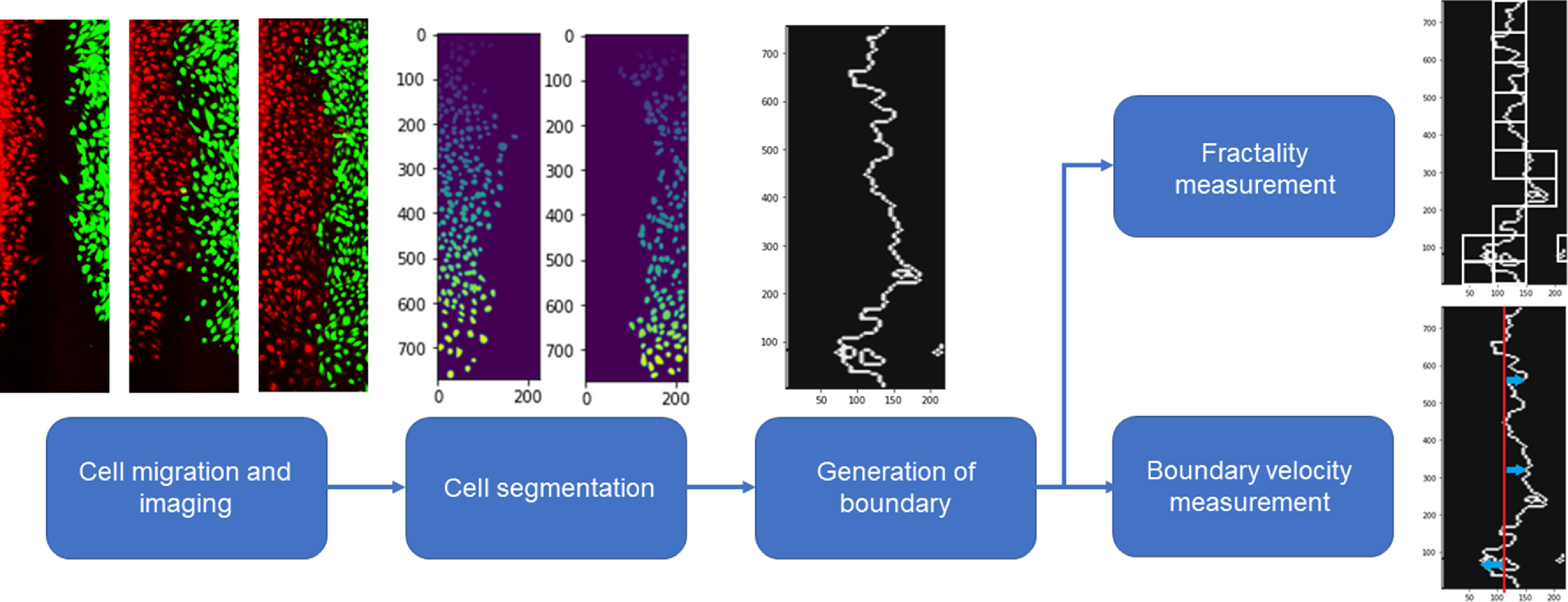
Schematic of the overall analysis. We take the time series fluorescent images of two groups of cells migrating toward each other and perform segmentation on the two channels. Then we generate the boundary between the two cell groups and calculate fractality of the boundary and the boundary velocity.

In this study, we propose the use of fractal dimension of the cell−cell boundary after two cell types are allowed to migrate towards each other (figure 1). Fractal dimension analysis has been widely used to study the geometric arrangements of various substances and materials, including tissues during development [30–33]. Some fractals are self-similar, meaning they exhibit geometric similarity at any scale, and there are several techniques and mathematical approaches that can be used to generate and describe these self-similar geometries [34]. However, many natural architectures do not show self-similarity, but instead exhibit a scale-limited similar pattern, making them pseudo-fractals [35]. These can also be analyzed using the same mathematical tools as self-similar fractals [35, 36].

Pseudo-fractals are commonly observed during the displacement of immiscible fluids in porous media, such as in enhanced oil recovery (EOR) processes [37, 38]. During EOR, capillary forces produce an interphase between the immiscible phases due to interfacial tension, velocity, and viscosity [39]. These forces can shape the boundary, resulting in straight and clear interphases or viscous fingering like patterns with pseudo-fractal behavior [40]. Similarly, the interaction of two different cell lines during tissue development is governed by differential affinity and altering this affinity can create different boundary patterns that can be analyzed using fractal dimension analysis. For example, in our previous study we demonstrated that surface receptor podoplanin is responsible for the distinct capillaries formed by blood endothelial cells (BECs) and lymphatic endothelial cells (LECs) [41]. To further explain the interaction between BECs and LECs, here we showed that fractal dimension analysis can be used to characterize BECs and LECs interactions during cell migration, an essential step toward blood and lymphatic tube formation [23, 42].

## 2. Methods

### 2.1. Cell segmentation and boundary definition

**Figure 2.**
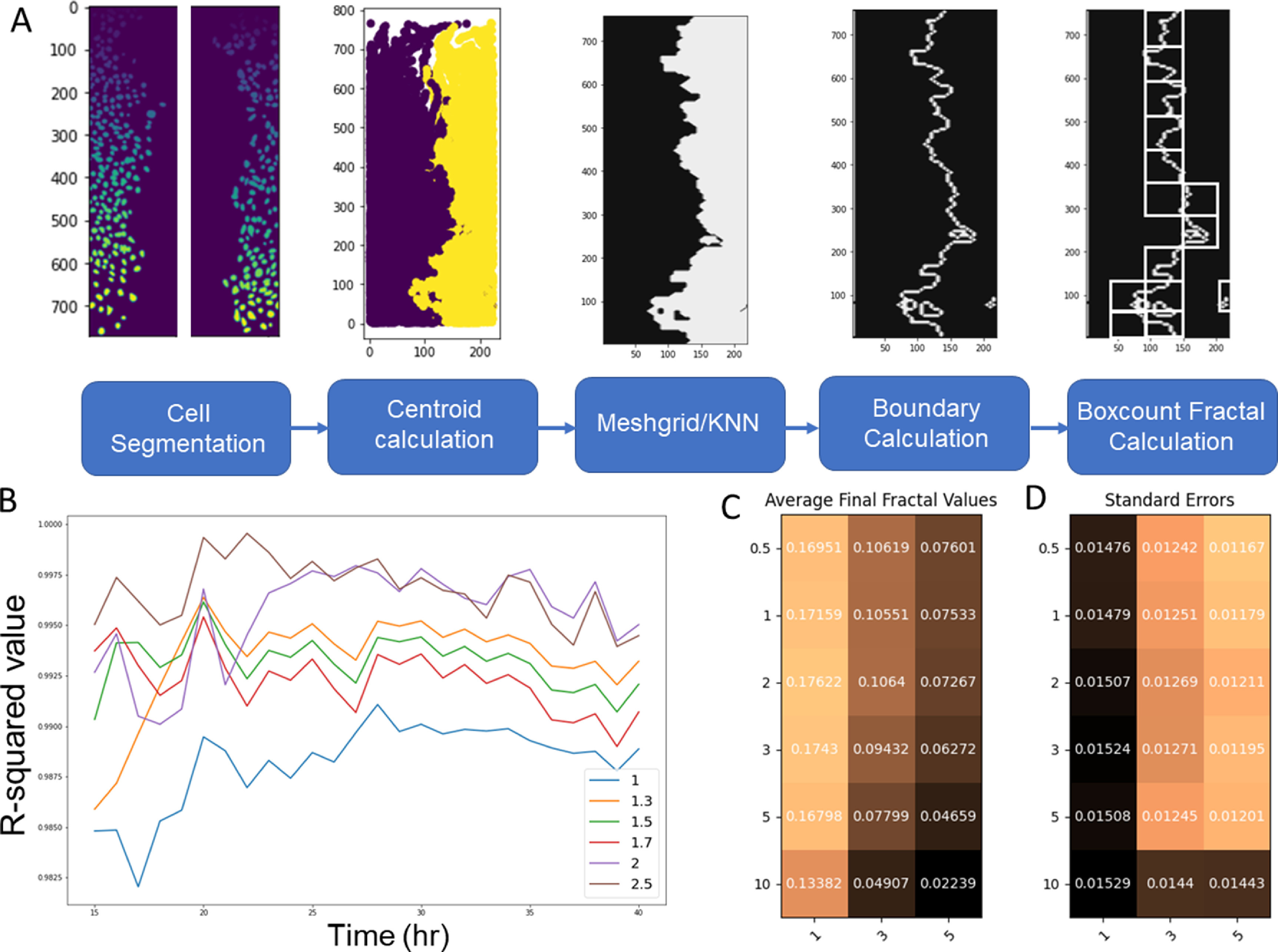
(A) Schematic of the algorithm and the optimization parameters. (B) Effect of the maximum box size in the box-counting algorithm on the *R* ^2^ value of the linear regression of log of box sizes and boundary-containing box counts. The maximum box size was defined as the number of pixels of the *x*-axis of the image divided by the box size parameter and rounded down to the nearest integer. (C) Optimization results based on the difference between the final fractal dimension estimate for LEC−LEC and BEC−BEC condition and LEC−BEC condition. (D) Optimization result based on the average standard error between the estimated fractality of each timeseries image of the same condition.

We utilized the data published by Jeong *et al* which included timeseries fluorescent images of BECs and LECs stained with different membrane staining dye and allowed to migrate towards each other [41]. We performed manual background subtraction by setting lower threshold to remove the background fluorescence. Videos over 25 MB in size were cropped into two separate videos to avoid problems associated with handling large file sizes. The timeseries images were segmented using Cellpose, a publicly available Python package that uses machine learning algorithm to segment the cells [43]. We used the Cyto model with an estimated diameter of 10 pixels to generate masks corresponding to the location of each cell for both types of cells. We then calculated the center of each mask of the cell to generate a single location point for each cell. The locations of cells for each timepoint were used as input data to generate the boundary. We generated a NumPy meshgrid of the same size as the input video and used SciPy’s K-Neighbor Classifier for each point to classify each point into either region 1 or 2 based on the number of cells nearest to it. Then we defined the boundary layer as the set of pixels in region 1 which bordered region 2 (figure 2(A)).

### 2.2. Box counting method

We determined the fractality of the boundary layer through box counting method. The image of the boundary layer, where the boundary pixels are assigned the value of 1 and non-boundary pixels 0, was used as the input to the algorithm. We generated a list of square box sizes from 2 pixels in length to half of the width of the video. Then we calculate the number of boxes that contain at least one border pixel for each box size, then plot the number of positive counts against the box sizes. We used NumPy’s polyfit function to generate the slope of the log−log values of box sizes and positive counts, which corresponds to the Hausdorff dimension of the boundary.

### 2.3. Boundary displacement calculations

We calculated the boundary layer velocity by estimating the average displacement of the boundary pixels from the initial position. The initial position was defined as the boundary layer when the cells first made contact, as visibly confirmed through fluorescent images. For each *y*-coordinate along the boundary, the absolute value of the deviation of the boundary layer from the initial position at that *y*-coordinate was averaged throughout the entire boundary. If there are multiple boundary layer pixels within a given *y*-coordinate, then the greatest value was taken. The average displacement was taken as the displacement of the boundary layer. To calculate the velocity, the boundary layer displacements were averaged over 10 most recent timepoints to smooth out the noise in the displacement.

### 2.4. Optimization of the algorithm

We optimized three parameters in our overall algorithm: the maximum box size in the box-counting algorithm, the coarseness of the NumPy meshgrid, and the number of neighbors during the K-nearest neighbors classification step. In generating the list of different sizes of boxes to use, we found that setting the largest box size to equal the width of the video resulted in box counts that deviated from the linear expectation. Therefore, we calculated the *R*-squared value of the linear fit resulting from box counting methods where the largest box size was set to the number of pixels of the *x*-axis of the image divided by box-size parameter *b* rounded to the nearest integer (figure 2(B)). We found that the optimum value of *b* was 2, meaning that the largest box size in our algorithm was half of the width of the video. We also determined the coarseness of the meshgrid by changing the number of points in the meshgrid each pixel would be represented by. We tested compression factors of 0.5, 1, 2, 3, 5, and 10, which correspond to the number of pixels each point in meshgrid represents. We also optimized for the different number of neighbors in the K-nearest neighbor algorithm. We used two metrics for optimizing these parameters: the difference in the average final fractal value between the LEC−LEC and BEC−LEC conditions and the average standard error for those two conditions. We want to maximize the difference in final fractal value and minimize the standard error to achieve the best algorithm that can discriminate between LEC−LEC and LEC−BEC condition while also yielding consistent results. Based on the results, we see that the meshgrid compression of 2 achieves optimal balance between the two metrics, and we observe a greater degree of discrimination and variability with lower number of neighbors (figures 2(C) and (D)). Therefore, for all further work, we used 1 nearest neighbor and meshgrid factor of 2.

### 2.5. Random walk simulation

We generated a NumPy array of 400 cells each of type 1 and 2, each consisting of *x*- and *y*-coordinate bound within 30 by 50 grid. The cells were initially organized in a block pattern, occupying 8 columns of 50 cells along the two edges of the grid. We designed a modified Gillespie algorithm by defining the relative probability of a cell moving into each of the four adjacent spaces. The simulation incorporated five parameters: *p_m_*_1_, *p_m_*_2_, *a_l_*_1_, *a_l_*_2_, and *a_d_*. Parameters *p_m_*_1_ and *p_m_*_2_ defined the probability of a cell moving within a given timeframe, and for all simulations they were set to 0.95 for both cell types. Parameters *a_l_*_1_ and *a_l_*_2_ defined the effect of a cell of the same type on the probability of the cell moving to the adjacent space respectively for cell type 1 and 2. Parameter *a_d_* defined the effect of the opposite cell type on the probability of cell movement. The cumulative effect of the like and dislike cells was calculated by multiplying the individual effects together. Therefore, *a* value greater than 1 indicates affinity between two cells, while *a* value of less than 1 indicates repulsion. We did not incorporate the impact of an immediately adjacent cell on the probability of the cell moving within a timepoint. A cell was forbidden from moving into a space that was already occupied by another cell or off the grid. For our simulation, we assumed no cell division or death. The overall probability of a cell of type 1 moving in direction *i* is therefore represented as

\begin{equation*}{p_{i,\,{\text{cell}}1}} = {p_{m1}}\left( {\frac{{{q_k}}}{{{{\mathop \sum \nolimits }}_{j = 0}^3\,{q_j}}}} \right)\end{equation*}

\begin{equation*} {q_j} = a_{l1}^{{n_l}}a_d^{{n_d}}r\end{equation*}

where *n_l_* and *n_d_* respectively represents the number of same and opposite type of cells adjacent to the space the cell might move to, and *r* is a binary value which is equal to 0 if the spot is occupied or exists at the edge, and 1 if available. Calculations for *q* was performed for each of the four spaces a cell can move into.

For each timepoint, the probability for each cell was calculated, and a random number generator determined the movement of each cell. For cells which could not move to any space in a given timepoint, the *q* values were all set to 0. The calculations were performed sequentially, where the previous cell was allowed to finish its movement before the probability was calculated for the next cell in order to prevent the probability of two cells moving into the same space. The order of the cells were randomly shuffled at each timepoint. The updated cell locations were saved as a NumPy array of *x*- and *y*-coordinates and used for subsequent fractal analysis using the same boundary defining and box counting methods described above.

## 3. Results

### 3.1. Lower differential affinity results in higher final fractality value

We used the data we previously published in the journal *Cellular Molecular and Bioengineering*, where we reported that podoplanin is responsible for the selective cell−cell interaction between BECs and LECs [41]. The LECs and BECs preferentially form tight junctions with only the cells of their own kind during capillary formation, allowing the separation of the blood and the lymph. We found that this recognition is dependent on the expression of podoplanin in LECs, which is consistent with previous observations [44–46]. Therefore, we found that podoplanin-knockout LECs (referred to as ΔLEC) act somewhat similarly to BECs and are recognized as pseudo-BECs.

**Figure 3.**
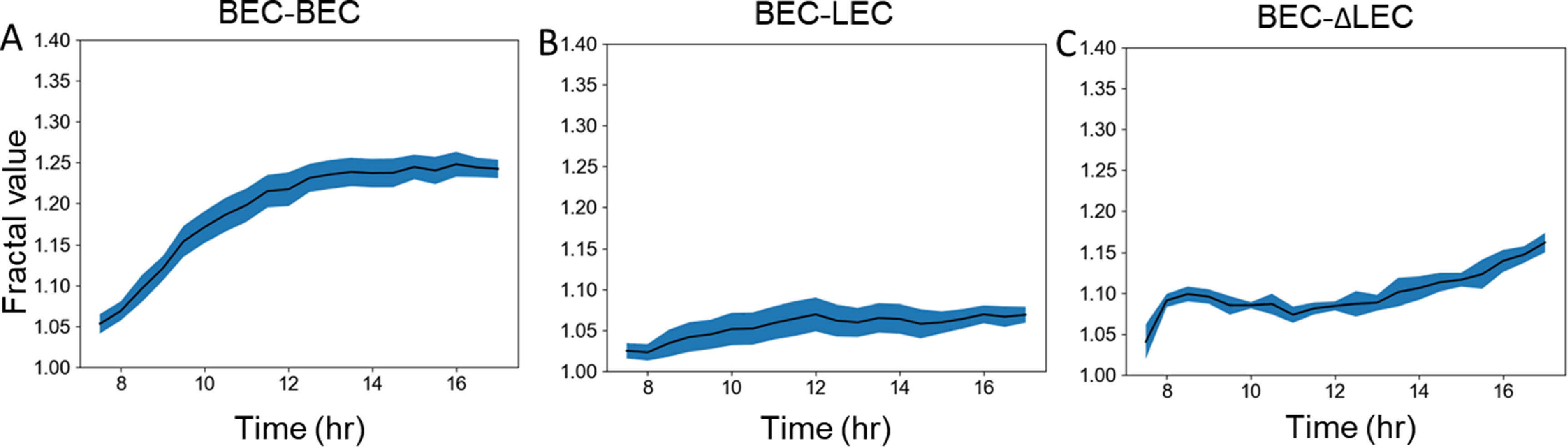
BEC and LEC migration fractality. Fractality of the (A) BEC−BEC, (B) BEC−LEC, and (D) BEC−ΔLEC conditions over time. Fractality was calculated after the gap had closed at around 8 h after the initial timepoint. Standard error is shown in blue. Standard error was calculated based on two experimental replicates which were segmented into smaller videos for ease of analysis.

As previously described, we performed migration assays with BECs and LECs stained in two different live cell tracking fluorescent dyes [41]. We seeded BECs on one side and either LECs, BECs, or ΔLECs on the other side and allowed the gap to close by cells migrating toward each other. We segmented the cell data and defined the boundary between the two groups of cells and calculated the fractality over time (figure 1). We found that the fractality of the boundary between two like cells, which exhibit no preference towards cells of its own side, increases to around 1.25, while the fractality of the two opposite cells remains around 1.05 (figures 3(A) and (B)). We only calculated the fractality after 8 h of migration when the gap fully closed, and the cells came into contact with each other. We also performed a similar analysis on the knockout condition and found the final fractal value to be around 1.15 (figure 3(C)). The fractal values correspond with the known affinity of BECs to other BECs versus LECs. We also expect the pseudo-LECs which express some BEC-like characteristics to have affinity for BECs that is between LECs and BECs, which also corresponds to the fractal values. However, it is challenging to measure the exact affinity of BECs to other BECs and LECs, which means the exact differential affinity is not measured. All *in vitro* cell migration assays were performed for 24 h at most, since beyond that point, cells will begin to undergo division and therefore the resulting fractality is not only a function of differential affinity, but also cell proliferative potential [42, 47].

### 3.2. Random walk simulation

**Figure 4.**
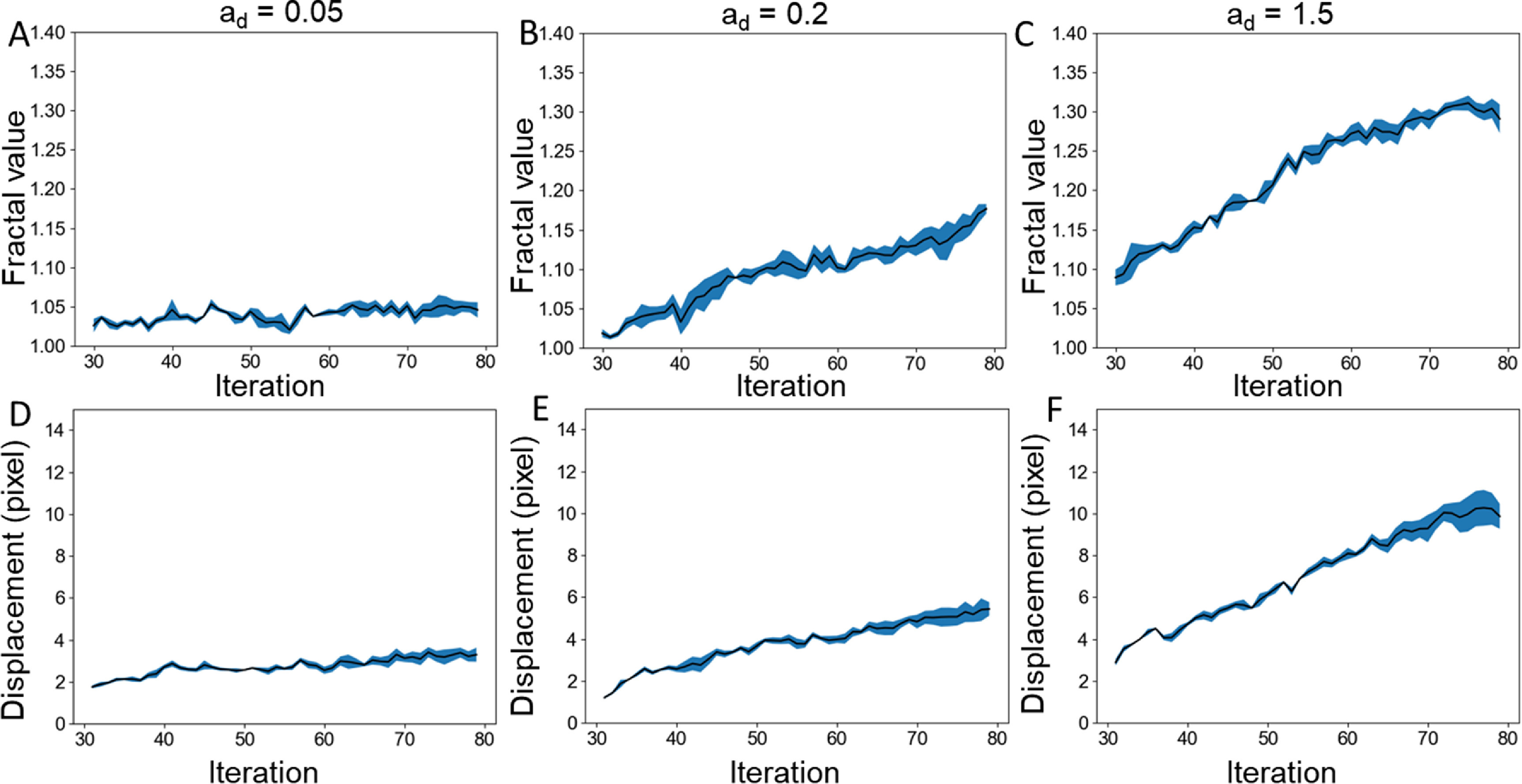
Random walk simulation results. Fractality of the (A) *a_d_* = 0.05, (B) *a_d_* = 0.2, and (C) *a_d_* = 1.5 conditions over time. The *p_m_* and *a_l_* values were kept constant at 0.95 and 1.5 respectively. Fractality was calculated after the gap had closed at around iteration 30. Boundary displacement calculations from the initial boundary position at iteration 30 with fractality of the (D) *a_d_* = 0.05, (E) *a_d_* = 0.2, and (F) *a_d_* = 1.5 conditions over time. Each condition was repeated three times. Standard error is shown in blue.

In order to measure the effect of differential affinity on fractality, we designed a random walk simulation to model the movement of the two types of cells. We tested five conditions: for all conditions, the probabilities of movement for both cells were set to 0.95, and the attractive force between the two like cells were set to 1.5 for both cells. For *a_d_* values, we tested 1.5 to simulate the BEC−BEC condition and 0.5, 0.2, 0.1, and 0.05 to simulate varying levels of differential affinity between BECs and LECs (supplemental figure 1). We ran the simulation for 80 timepoints and found that the gap is mostly closed by timepoint 30. We have empirically determined that 80 timepoints is the minimum timepoints required for all tested conditions to reach approximately zero boundary velocity when averaged over 5 consecutive timepoints. From timepoint 30–80, we performed the fractality analysis and found that greater differential affinity results in significantly lower fractality value (figures 4(A)–(C)). For *a_d_* value of 1.5, the fractal value reached close to 1.30, similarly to our BEC−BEC condition, and for 0.05, the fractal value remained close to 1.05, analogous to BEC−LEC condition. For *a_d_* value of 0.2, the fractal dimension reached approximately 1.15 similarly to our BEC−ΔLEC condition. We also calculated the boundary layer displacement for each of these conditions and found that higher differential adhesion led to lower boundary layer displacement as expected (figures 4(D)–(F)).

### 3.3. Relationship with the capillary number

To determine if our analysis of the fractal dimension is analogous to viscous fingering, we analyzed the relationship between capillary number and fractality in our simulations. It has been previously reported that in viscous fingering, *Ca* is negatively correlated with fractality [48]. In biological applications, unlike in petroleum engineering, the capillary number does not vary by large magnitudes, therefore, we assume local linear relationship between fractality and capillary number

\begin{equation*}f = - mCa + b = - m\frac{{\mu V}}{\sigma } + b\end{equation*}

where *V* represents boundary velocity, *σ* is the interfacial tension, *µ* is the viscosity, and *m* and *b* represents constants. We expected to see that the fractality of the boundary would be close to 1 when the cells are moving rapidly to close the gap, which is when the cells are moving at their maximum speed in absence of obstacles. At point of initial contact, when the boundary is essentially a one-dimensional straight line, the velocity would also equal *V*_max_ if we assume continuity of velocity. Effectively, we observe that boundary velocity acts as a proxy for time, transitioning from initial *V*_max_ to final velocity of 0 as cells lose the driving force behind migration as the cell density reaches equilibrium. Therefore, we expected fractal value to be close to 1 at maximum velocity, which is at initial timepoint, which allowed us to make the following prediction:

\begin{equation*}\mathop {\lim }\limits_{V \to {V_{{\text{max}}}}} f = 1.\end{equation*}

Likewise, we can assume that the viscous fingering will reach equilibrium at distant timepoint, which is when the boundary velocity will reach 0. At this point, the fractality would no longer change, meaning this would be our final fractal value. Therefore, we generate another prediction:

\begin{equation*}\mathop {\lim }\limits_{V \to 0} f = {f_{{\text{final}}}}.\end{equation*}

Therefore, we see that if for all conditions, as *V* approaches *V*_max_ the fractality must approach 1. This means we can express fractality as

\begin{equation*}f = - m\frac{{\mu V}}{\sigma } + 1 + m\frac{{\mu {V_{{\text{max}}}}}}{\sigma },\end{equation*}

where *m* is a constant. We can therefore predict that the final fractal value will have the following relationship:

\begin{equation*}{f_{{\text{final}}}} = m\frac{{\mu {V_{{\text{max}}}}}}{\sigma } + 1 \propto \frac{1}{\sigma }.\end{equation*}

We also predicted that the rate of change of fractality with velocity to be approximately

\begin{equation*} - \frac{{{\text{d}}f}}{{{\text{d}}V}} = m\frac{\mu }{\sigma } \propto \frac{1}{\sigma }.\end{equation*}

Based on this, we estimated that the final fractal value would be inversely proportional to the differential affinity or surface tension, and that the rate of change of fractality with velocity would be negatively correlated with differential affinity. Using the simulated cells, we found that these relationships are indeed shown to be true. For the model parameters, we held constant the viscosity *μ,* analogous to the *p_m_*_1,2_ parameter in our model, and estimated that interfacial tension *σ* would be analogous to *a_l_*/*a_d_*. This means that lower *a_d_* value indicates stronger repulsion by the other cell type and therefore results in higher surface tension. In our simulations, we found that both the final fractal value and the slope of fractal-velocity plot were both inversely proportional to *σ*. We fit the data we derived from the random walk simulations with inverse proportion equation through non-linear least squares fitting method and derived the following relations:

\begin{equation*}{f_{{\text{final}}}} = \frac{{1.2737}}{{\sigma + 3.7595}} + 1\end{equation*}

\begin{equation*} - \frac{{{\text{d}}f}}{{{\text{d}}V}} = \frac{{0.7181}}{{\sigma + 2.1533}} + 0.0447.\end{equation*}

**Figure 5.**
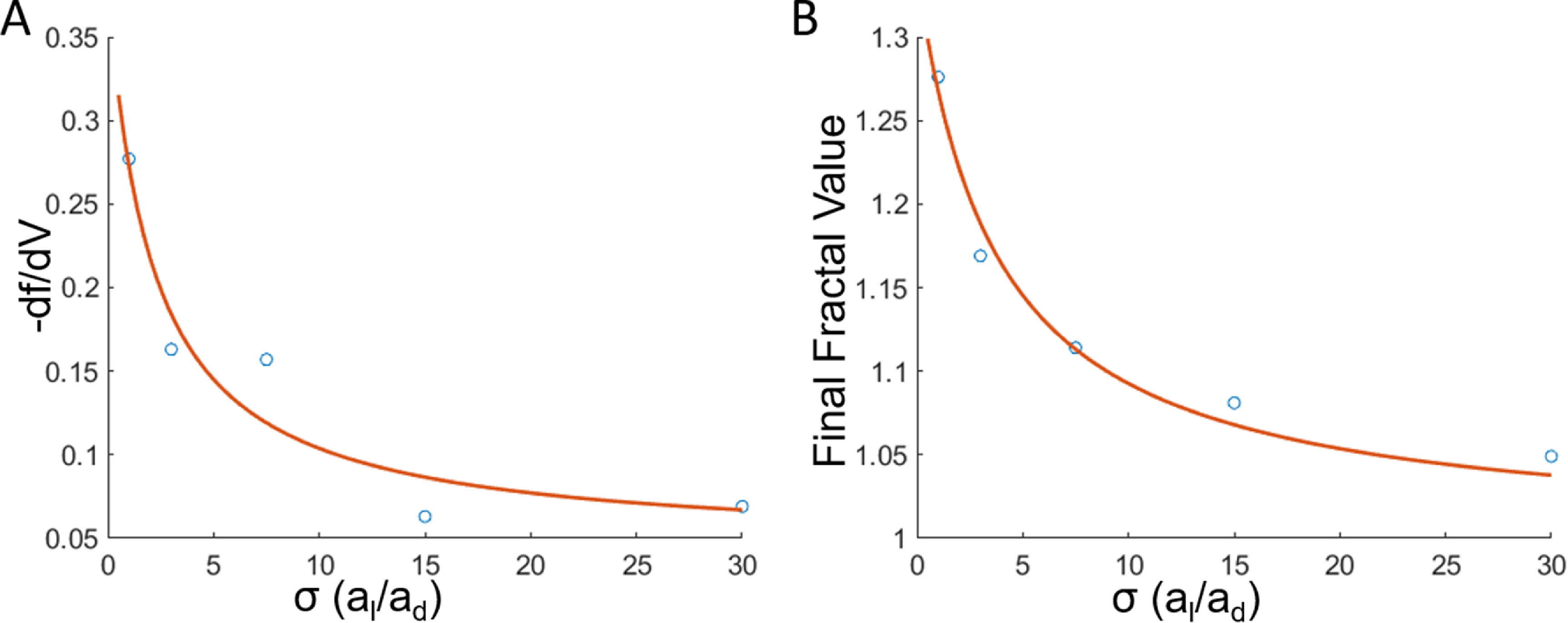
The relationship between *σ* and fractality/velocity. (A) Plot of the model estimate of interfacial tension versus the absolute value of rate of change of fractal dimension of the boundary with respect to the boundary velocity. (B) Plot of the model estimate of interfacial tension versus the final fractal value at iteration 80 in the simulations. The inverse function fit using nonlinear least squares curve fitting method is shown in red. Five conditions were tested at various ad values while *a_l_* and *p_m_* were held constant.

We achieved the square norm of the residual values of 0.0007 and 0.0025 respectively for the *σ* vs final fractal value and *σ* vs negative of slope values (figures 5(A) and (B)). To confirm that the calculated values are best fit by the inverse function, we also fit the data to linear, second-order polynomial, exponential decay and confirmed that the inverse function yields the lowest norm of the residual (supplemental figure 2). These results indicate that our fractal analysis responds similarly to fractality of viscous fingering systems. Since there is a strong inverse relationship between the final fractal value and the interfacial tension, this suggests that fractal dimension measurement of the cell−cell boundary can be used as a relative measure of cell−cell adhesion.

## 4. Discussion

In this study, we have used fractality of the boundary between two types of cells to estimate the differential affinity between them. We modeled the cells as a system consisting of two partially miscible fluids allowed to move toward each other in a 2D surface, which can result in viscous fingering pattern. It is important to note that as the cells from single monolayers, we do not expect the vertical dimension to affect our conclusion. While random walk simulations can easily be modified to be run in 3D, the agreement in fractal dimension with the 2D imaging suggests that a 2D model is sufficient. Similar to how the fractal dimension of the fingering pattern is correlated with the capillary number, we have determined that the fractality of the fingering-like patterns visible in the cell migration patterns also follow the similar relationship between fractality and parameters analogous to capillary number [36]. Therefore, we estimate that the differential affinity, which is roughly analogous to the interfacial tension in a fluid−fluid boundary, is inversely correlated with the fractal dimension of the cell−cell boundary layer.

Our findings suggest that the differential affinity between cells of different origins play a crucial role in cell organization and boundary formation, which seems to suggest differential adhesion hypothesis (DAH) first introduced by Twones and Holtfreter in their landmark 1955 study [49]. Since then, numerous studies have reported on the effects of differential adhesion on the formation of cell aggregate boundaries, which can be modeled to be liquid-like. Varying the cadherin levels in L cell aggregates have affected the segregation and sorting of cells guided by free energy of cell−cell binding [9]. In another study, Toll1, a transmembrane protein that plays a role in intercellular adhesion, has been shown to correct distortions in *Drosophila* pupa epidermal epithelium, further supporting the DAH [50]. However, DAH has been a controversial subject in the field of tissue engineering and developmental biology, with some studies disputing the validity of modeling cell aggregates as immiscible fluids and suggesting dynamic factors in addition to differential adhesion may play a significant role in cell organization [51, 52]. Our findings endorses the validity of DAH in the self-organizing behavior of BECs and LECs as well as put forth a new mathematical approach to model the cell−cell boundary behavior. Future studies should explore the use of various grid coarseness in our random walk simulations to fully confirm that the cell−cell boundary can be modeled accurately as a continuum similarly to partially miscible fluids despite the significantly larger size of each ‘particle’.

Overall, our findings indicate that the fractality measurement can be a simple tool to estimate the differential affinity in two groups of cells, which is useful in co-cultured tissue engineering where two or more types of cells must self-organize to form structures [2, 22]. Future studies could explore the use of this tool in various types of cells that may exhibit differential affinity, for example in tumor cells that attract endothelial cells for vessel formation [53, 54]. Our random walk simulation model could also be applied to predict the formation of blood vessels in vascularized organoids, where mobile endothelial cells invade a less mobile group of cells to form perfusing vessels [55, 56]. Taken together, our fractality tool is a novel approach to measure the differential affinity between cells that does not require protein level analysis, which can have a broad application in cell development and tissue engineering.

Future studies should explore the limitations and potential sources of error in the methods used in this study. While box-counting method is a widely used method for estimating fractal dimensions, it suffers from quantization error, which stems from variabilities in grid orientation and placement as well as scaling range [57]. In order to reduce the effect of the errors in box-counting method, we determined the linear region over various box scaling ranges, but improvements on the box counting method such as pattern search which have higher computational cost could be applied in future studies [58]. Additionally, we included five variable parameters in the random walk simulations in order to potentially model the variabilities in cell motility characteristics, but for the simplicity of our mathematical derivations we only tested the effect of varying *a_d_* which subsequently varies our simulated interfacial tension. Future studies using our random walk simulation could explore the effects of varying migration potential and the *a_l_* parameters to fully capture the dynamics between various cell lines.

## Data Availability

The data that support the findings of this study are openly available at the following URL/DOI: https://github.com/donghyunjeong21/FractalityOfCellBoundary.
